# Procrastination in Daily Working Life: A Diary Study on Within-Person Processes That Link Work Characteristics to Workplace Procrastination

**DOI:** 10.3389/fpsyg.2018.01087

**Published:** 2018-07-05

**Authors:** Roman Prem, Tabea E. Scheel, Oliver Weigelt, Katja Hoffmann, Christian Korunka

**Affiliations:** ^1^Department of Applied Psychology: Work, Education, Economy, Faculty of Psychology, University of Vienna, Vienna, Austria; ^2^Faculty of Informatics, Communication and Media, University of Applied Sciences Upper Austria, Hagenberg im Mühlkreis, Austria; ^3^Department of Occupational and Organizational Psychology, International Institute of Management and Economic Education, Europa-Universitaet Flensburg, Flensburg, Germany; ^4^Department of Organizational and Personnel Psychology, Institute of Management, Faculty of Economic and Social Sciences, University of Rostock, Rostock, Germany; ^5^Department of Work and Organizational Psychology, Institute of Psychology, Faculty of Humanities and Social Sciences, FernUniversität in Hagen, Hagen, Germany

**Keywords:** workplace procrastination, self-regulation, challenge-hindrance, time pressure, planning and decision-making, problem solving, diary study

## Abstract

Procrastination is a form of self-regulation failure characterized by the irrational delay of tasks despite potentially negative consequences. Previous research on procrastination was mainly conducted in academic settings, oftentimes combined with a focus on individual differences. As a consequence, scholarly knowledge about how situational factors affect procrastination in work settings is still scarce. Drawing on job stress literature, we assumed that work characteristics go along with cognitive appraisals of the work situation as a challenge and/or hindrance, that these cognitive appraisals affect employees' self-regulation effort to overcome inner resistances, and that self-regulation effort should in turn be related to workplace procrastination. In our study, we focused on three specific work characteristics that we expected to trigger both challenge and hindrance appraisal simultaneously: time pressure, problem solving, and planning and decision-making. We hypothesized serial indirect effects of these work characteristics on workplace procrastination via cognitive appraisal and self-regulation processes that unfold within individuals over short periods of time. Consequently, we conducted a diary study with three measurement occasions per workday over a period of 12 days. Overall, 762 day-level datasets from 110 employees were included in Bayesian multilevel structural equation modeling (MSEM; controlled for sleep quality and occupational self-efficacy). Our results revealed negative serial indirect effects of all three work characteristics on workplace procrastination via increased challenge appraisal and subsequently reduced self-regulation effort. Further, our results showed a positive serial indirect effect of time pressure (but not of problem solving or planning and decision making) on workplace procrastination via increased hindrance appraisal and subsequently increased self-regulation effort. Overall, our study showed that work characteristics are linked to workplace procrastination via within-person processes of cognitive appraisal and self-regulation. Because not all work characteristics triggered hindrance appraisal, we argue that it may make sense to further differentiate challenge stressors in the future. Moreover, cognitive appraisals affected self-regulation effort only on the within-person level. On the between-person level self-regulation effort was strongly negatively related with occupational self-efficacy. Thus, we conclude that depending the perspective on procrastination (e.g., differential psychology perspective vs. situational perspective) different variables will be considered relevant to explain the emergence of procrastination.

## Introduction

Procrastination is a form of self-regulation failure that is characterized by the needless delay of things one intends to do despite the expectation of negative consequences (Steel, 2007; cf. Klingsieck, 2013). It has been estimated that the majority of college students engage in procrastination and consider themselves procrastinators, but also that about one in five adults are chronically affected by procrastination (cf. Steel, 2007). Chronic procrastinators perform more poorly overall and feel more miserable in the long term (Steel, 2007). Regarding the work domain, it has been reported that employees spend about 90–180 min per workday on personal activities (potentially including procrastination) during their working hours (Paulsen, 2015). The annual loss per employee due to personal activities during working hours is estimated at $8,875 (D'Abate and Eddy, 2007).

Despite the prevalence and relevance of procrastination in work settings, most of previous research on procrastination was conducted in academic settings (van Eerde, 2016). Thus far, research on workplace procrastination has investigated how chronic procrastinators are evaluated (Ferrari, 1992), which jobs chronic procrastinators occupy (Nguyen et al., 2013), and to which extent different types of time perspective predict the tendency to procrastinate in the workplace (Gupta et al., 2012). Empirical studies focusing on situational factors as antecedents of workplace procrastination investigated the relationships of work characteristics with decisional procrastination at work (Lonergan and Maher, 2000) and workplace procrastination in general (Metin et al., 2016). However, because most studies on workplace procrastination were based on cross-sectional study designs that do now allow to draw conclusions about within-person effects, knowledge about how situational factors lead to workplace procrastination is still scarce.

In this paper, we focus on procrastination in work settings and examine within-person processes that link work characteristics to workplace procrastination. We draw on literature regarding self-regulation at work (MacKey and Perrewé, 2014) to explain how effortful self-regulation might link work characteristics to self-regulation failure in the form of workplace procrastination. We further investigate how employees' cognitive appraisals of the work situation might impact on their self-regulation effort and thus affect daily workplace procrastination. Given our assumption that these within-person processes of cognitive appraisal and self-regulation effort unfold over rather short periods of time, we conducted a diary study with multiple measurement occasions per workday.

Our research aims to advance the academic literature on procrastination in multiple ways. First, our study investigates procrastination in the work domain to extend the research on procrastination in non-academic settings. Second, following traditional approaches in job stress research, our study considers work characteristics as antecedents of occupational behavior and thus considers situational factors as antecedents of procrastination. Third, with the implementation of a diary study the design of our study goes beyond cross-sectional designs traditionally used in procrastination research. Using a diary study design allowed us to investigate within-person processes of cognitive appraisal and self-regulation effort in daily working life where they unfold. This enables our study to shed light on the within-person processes that link situational factors to workplace procrastination.

### Perspectives on procrastination and procrastination in daily working life

Explanations for the emergence of procrastination differ depending on the standpoint scholars take. From a differential psychology perspective, procrastination is a trait that is associated with other personality variables. From a motivational and/or volitional psychology perspective, procrastination is a motivational and/or volitional deficit that is associated with other motivational and self-regulation variables. From a clinical psychology perspective, procrastination is a clinically relevant phenomenon that is associated with anxiety, depression, and stress. Finally, from a situational perspective, procrastination is evoked by certain situational features like task difficulty (Klingsieck, 2013).

In this paper we will approach procrastination from two of these perspectives, that is, from a situational as well as a motivational/volitional perspective. Following traditional job stress research, we investigate the impact of work characteristics on procrastination at work and hence take a situational perspective. Additionally, we also draw on literature on self-regulation at work (MacKey and Perrewé, 2014) that combines cognitive appraisals and self-regulation effort to explain work behavior. Thus, we also take a motivational/volitional perspective to explain procrastination.

Our focus on the situation and rather short-term within-person processes to explain workplace procrastination suppose that the level of workplace procrastination fluctuates in daily working life. In recent diary studies, it has been shown that workplace procrastination indeed shows meaningful within-person fluctuations (Kühnel et al., 2016, 2018; regarding fluctuations in daily task completion also refer to Claessens et al., 2010). Thus, it is safe to assume that employees' abilities to initiate and complete actions vary not only between individuals at a given point in time but also within individuals over time. In other words, a specific employee might not only procrastinate more or less on average than other employees on average, she/he might also procrastinate more or less on a given day than on other days.

### Within-person processes that link work characteristics to workplace procrastination

In the following, we will draw on the appraisals, attributions, adaptation (AAA) model of job stress (MacKey and Perrewé, 2014) that integrates various theories to describe how situational factors affect emotions and individual coping behaviors at work via cognitive appraisal and self-regulation processes. The AAA model of job stress builds upon the transactional theory of stress (Lazarus and Folkman, 1984) and its extension (Perrewé and Zellars, 1999), as well as self-regulation theories (Muraven and Baumeister, 2000). By considering the impact of cognitive appraisals on self-regulation processes, the AAA could provide considerable insights into the underlying mechanisms of within-person processes that link job stressors to workplace procrastination.

Based on previous research, the AAA model differentiates between challenge stressors and hindrance stressors (e.g., LePine et al., 2005). Challenge stressors include work characteristics like workload, responsibility, and job complexity, whereas hindrance stressors include work characteristics like role ambiguity, role conflict, and red tape (LePine et al., 2005). Challenge stressors and hindrance stressors both lead to employee strain, but only challenge stressors can also boost employees' motivation and thus have more favorable effects on employee well-being and performance than hindrance stressors (e.g., LePine et al., 2005; Crawford et al., 2010; Prem et al., 2018). Because challenge stressors lead to both, strain as well as motivation and well-being, they oftentimes show ambivalent relationships with performance-related work outcomes (LePine et al., 2005). Thus, we assume that challenge stressors may trigger both, adverse and favorable processes that affect workplace procrastination.

It has indeed been shown that challenge stressors usually promote both challenge appraisal and hindrance appraisal simultaneously and that these appraisals explain the effects of job stressors on work outcomes (e.g., Webster et al., 2011; Searle and Auton, 2015). The cognitive appraisal of a work situation as more challenging and/or more hindering will elicit specific positive and/or negative emotions (Perrewé and Zellars, 1999) that entrain action tendencies (MacKey and Perrewé, 2014). For example, a person feeling more negative emotions (or less positive emotions) as a consequence of higher hindrance appraisal (or lower challenge appraisal) might have a tendency to withdraw from the situation that may be perceived as an inner resistance toward the work tasks. To overcome their inner resistances employees have to suppress their action tendencies and alter their coping behavior through effortful self-regulation (Muraven and Baumeister, 2000). However, with increasing effort required to overcome inner resistances it will become more likely that self-regulation fails, and thus, that workplace procrastination will be more likely to occur when self-regulation effort is higher.

The idea that emotions elicited in cognitive appraisal processes may entrain action tendencies to withdraw from the situation and thus promote procrastination is also compatible with recent literature that describes procrastination as an emotion-regulation strategy that provides short-term mood repair (Sirois and Pychyl, 2013; Pychyl and Sirois, 2016). It is assumed that self-regulation failure to initiate and/or complete an action in a specific situation may result from a person failing to inhibit hedonistic impulses to switch to more instantly gratifying activities. Thus, procrastination can also be depicted as a maladaptive emotion-focused coping strategy where individuals try to attain a hedonic shift to get out of negative emotions (Pychyl and Sirois, 2016).

**Figure 1:**
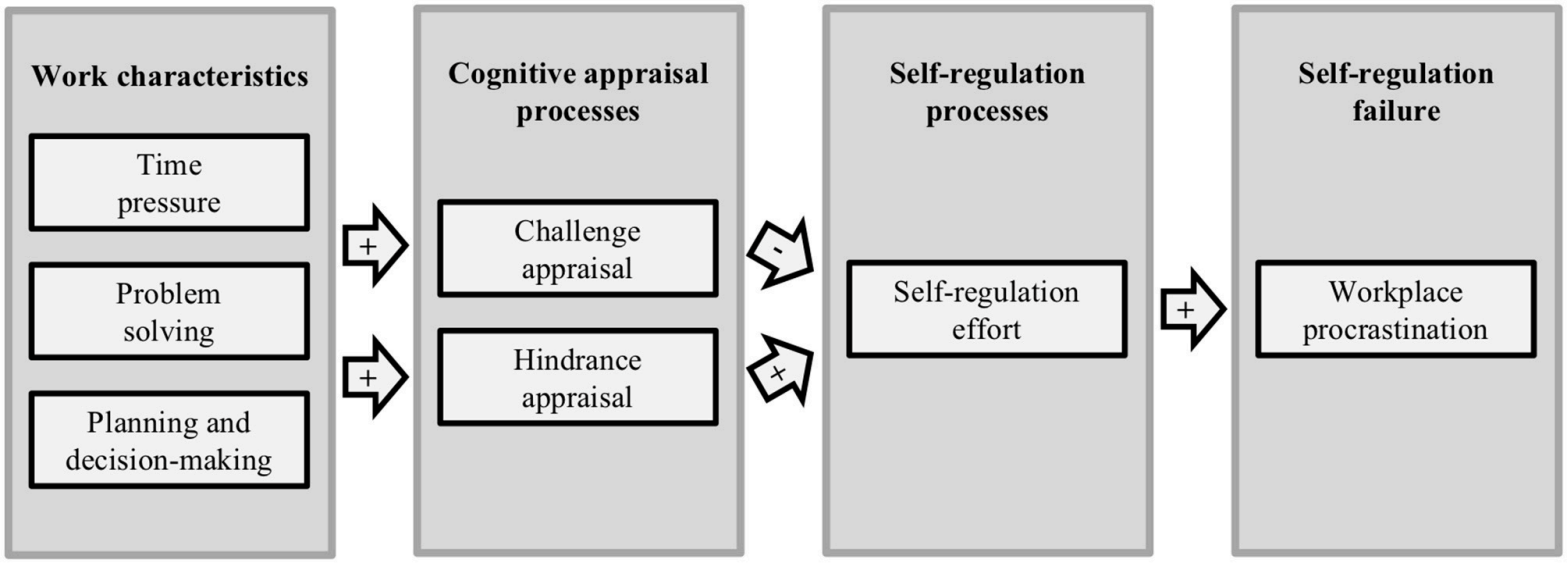
Conceptual model of the study regarding serial within-person effects of work characteristics on workplace procrastination via cognitive appraisals and self-regulation effort.

Drawing on the AAA model of job stress and the literature on procrastination as an emotion-regulation strategy, we assume that the extent to which work characteristics promote challenge and/or hindrance appraisal explains how much effort will be necessary in self-regulation processes to overcome inner resistances and that higher self-regulation effort will in turn go along with increased levels of workplace procrastination. Figure 1 gives an overview of the conceptual model of our study.

We will test our assumptions for three specific work characteristics that we expect to trigger both challenge and hindrance appraisals in daily working life: time pressure, problem solving, and planning and decision-making. Time pressure is an indicator of high quantitative demands and can be described as the extent to which employees feel that they need to work at a faster than usual pace or have insufficient time to finish their work tasks (Kinicki and Vecchio, 1994; Baer and Oldham, 2006). Problem solving, on the other hand, is an indicator for qualitative demands and can be described as the degree to which a job requires unique ideas or solutions and reflects the more active cognitive processing requirements of a job (Morgeson and Humphrey, 2006). Finally, planning and decision-making refers to requirements that employees plan and structure their workday, determine how to handle their work tasks, and decide on the priority of work tasks on their own (Kubicek et al., 2015). Given that the appraisal of work characteristics as a challenge should reduce self-regulation effort and the appraisal as a hindrance should increase self-regulation effort, we propose:

Hypothesis 1: Day-level work characteristics, i.e., (a) time pressure, (b) problem solving, and (c) planning and decision-making, have a negative serial indirect effect on daily workplace procrastination via increased challenge appraisal and consequently reduced self-regulation effort.
Hypothesis 2: Day-level work characteristics, i.e., (a) time pressure, (b) problem solving, and (c) planning and decision-making, have a positive serial indirect effect on daily workplace procrastination via increased hindrance appraisal and consequently increased self-regulation effort.

## Method

### Participants and procedure

As our study focused on within-person processes that link work characteristics to workplace procrastination, we decided to recruit employees to participate in a diary study (Ohly et al., 2010; Fisher and To, 2012). The sample consisted of employees with regular employment enrolled in a distance learning undergraduate psychology program of a German university. This population is more similar to the general working population than to typical undergraduate samples (e.g., Dabbagh, 2007; Syrek et al., 2017).

This study was carried out in accordance with the recommendations of the American Psychological Association (2010). The procedure and materials of this study have not undergone examination by an ethics committee, as the measures and procedures of our study followed the protocols of standard diary study research in applied psychology and we did not touch sensitive topics (like e.g., sexual orientation). Our protocol fully complied with the standards of the university where it was conducted (i.e., shared affiliation of third and fourth author). These standards include strict guidelines to store potentially identifying information like e-mail addresses separately from the focal measures. Individuals interested in participating in our study were informed about the general aims and the protocol of the study before their participation. Participation was voluntary and participants had the opportunity to quit whenever they wanted. The announcement of the study, along with an e-mail assuring confidentiality and voluntary participation, was sent to all employees, who had given their e-mail address confirming their interest to participate in the study. Study participants could earn required study credits as research participants.

Participants were asked to complete a general survey and multiple daily surveys. The general survey had to be filled out before starting the daily surveys. Participants were instructed to complete the daily surveys over a period of 12 consecutive days starting on a Friday and ending on a Tuesday. Our study also included measurements on the weekend because (a) we were not sure beforehand whether some participants might be working on Saturdays and/or Sundays and (b) we asked participants to answer questions regarding their recovery during their days off to answer research questions not relevant for the present paper. On workdays participants were asked to fill out diary entries three times a day: (1) in the morning before starting work, (2) in the afternoon after lunchbreak, and (3) in the evening at the end of the working day.

The general survey was completed by 130 individuals, who provided a total of 1,272 out of 1,560 possible day-level datasets (130 participants × 12 days). This means that participants provided diary entries on average on 9.78 out of the 12 days and that the compliance of filling out the diary was satisfactory (81.5%). We excluded day-level datasets from our analyses if participants had indicated that they were not working on the respective day. After this step, 786 day-level datasets from 122 individuals remained. We further excluded datasets from our analyses if participants had failed to provide at least 3 day-level datasets. Overall, 762 day-level datasets from 110 individuals could be used in the analyses.

As 20 participants, who completed the general survey but failed to provide day-level datasets on at least 3 working days, were excluded from the final sample, we conducted a binary logistic regression analysis to see if sociodemographic information from the general survey (gender, age, job tenure, working time per week, permanent contract, working full time, leadership position) could be used to predict whether a participant would be included in the final sample or not. None of the variables emerged as a significant predictor in the binary logistic regression analysis. This suggests that the attrition in our sample was not systematic with regard to sociodemographic variables.

In the final sample (77.3% female), the mean age was 35.1 years (*SD* = 10.0); mean job tenure was 12.6 years (*SD* = 10.0); mean working time was 33.9 h per week (*SD* = 11.4). Employees came from different branches (industry 8%, marketing and sales 5%, consulting and finance 5%, health care and social services 28%, information technology 5%, public relations 3%, other services 7%, public administration 8%, research and development 7%, and other branches 24%). Most employees had a permanent contract (71%), worked full time (56%), and almost one third (31%) had a leadership position.

### Measures

As in most diary study research, we used abbreviated scales for all measures to reduce participants' burden of filling out long scales multiple times (Ohly et al., 2010; Fisher and To, 2012). Participants were instructed to answer all items with regard to their current workday. All items were administered in German on 5-point scales (1 = *strongly disagree*; 5 = *strongly agree*; except for sleep quality).

*Time pressure* was assessed in the afternoon after lunchbreak with three items adapted from the instrument for stress-oriented job analysis (Semmer et al., 1999; see also Prem et al., 2017). A sample item is “This morning, I was pressed for time.”

*Problem solving* was measured in the afternoon after lunchbreak with three items adapted from the Work Design Questionnaire (Morgeson and Humphrey, 2006; see also Stegmann et al., 2010). A sample item is “This morning, my job involved solving problems that have no obvious correct answer.”

*Planning and decision-making* was assessed in the afternoon after lunchbreak with three items adapted from the Intensification of Job Demands Scale (Kubicek et al., 2015; see also Prem et al., 2016). A sample item is “This morning, my job required me to make decisions on the priority of tasks on my own.”

*Challenge appraisal* was measured in the afternoon after lunchbreak with four items adapted from Searle and Auton (2015). A sample item is “This morning's situations and events will help me to learn a lot.”

*Hindrance appraisal* was measured in the afternoon after lunchbreak with four items adapted from Searle and Auton (2015). A sample item is “This morning's situations and events will restrict my capabilities.”

*Self-regulation effort* was assessed in the evening at the end of the working day with three items adapted from the overcoming inner resistances subscale by Schmidt and Neubach (2010) (see also van Hooff and Geurts, 2015). A sample item is “Today, starting certain tasks required me to use a lot of willpower.”

*Workplace procrastination* was measured in the evening at the end of the working day with six items adapted from Tuckman (1991) (see also Kühnel et al., 2016, 2018). A sample item is “Today, I needlessly delayed finishing jobs, even when they were important.”

We controlled for sleep quality and occupational self-efficacy in our analyses based on previous research on workplace procrastination (Prem et al., 2015; Kühnel et al., 2016, 2018). *Sleep quality* was assessed in the morning before starting work with four items adapted from the Insomnia Severity Index (Bastien et al., 2001). A sample item is “How satisfied are you with your last night's sleep pattern?” (1 = *very satisfied*; 5 = *very unsatisfied*, recoded). *Occupational self-efficacy* was also measured in the morning before starting work with five items adapted from Rigotti et al. (2008). A sample item is “Whatever comes my way in my job today, I can handle it.”

To show that the variables measured in the daily diary represent empirically distinct constructs, we conducted multilevel confirmatory factor analyses (MCFAs) with Mplus 8 (Muthén and Muthén, 1998-2017). The MCFAs showed a satisfactory fit of the hypothesized nine-factor model (χ^2^ = 1939.9, *df* = 1048, RMSEA = 0.03, CFI = 0.93, TLI = 0.92, AIC = 46654.4) where the items of each measured construct were set to load on the factor of their respective construct only. We compared this hypothesized 9-factor model against a total of 36 different eight-factor models where the items of two constructs were set to load on a common factor. If one of these models were superior to the hypothesized nine-factor model, this would indicate that the items set to load on the same factor measure the same latent construct. As the best-fitting of all 36 eight-factor models (i.e., the model with hindrance appraisal and sleep quality on the same factor; χ^2^ = 2527.0, *df* = 1064, RMSEA = 0.04, CFI = 0.89, TLI = 0.88, AIC = 47119.6) was not superior to the hypothesized nine-factor model, this indicates that all scales measured empirically different constructs. We further tested the hypothesized nine-factor model against a three-factor model with all items measured at the same measurement occasion loading on a common factor (χ^2^ = 7602.1, *df* = 1114, RMSEA = 0.09, CFI = 0.51, TLI = 0.47, AIC = 52094.7) and the one-factor model with all items loading on a single factor (χ^2^ = 10797.3, *df* = 1120, RMSEA = 0.11, CFI = 0.26, TLI = 0.22, AIC = 55277.9). As the hypothesized nine-factor model fitted better than any of the alternative models, it can be concluded that the variables measured in the daily diary represent empirically distinct constructs.

### Data analysis

Because our data had a nested data structure with day-level datasets nested within individuals, we tested our hypotheses using multilevel structural equation modeling (MSEM; Zhang et al., 2009; Preacher et al., 2010, 2011) in Mplus 8 (Muthén and Muthén, 1998-2017). By decomposing the variance of variables into their between-person and within-person components, MSEM accounts for the fact that relationships might be different on the between-person and the within-person levels. Thus, multilevel mediation analyses with MSEM are less prone to biases than other techniques of multilevel mediation analysis (Zhang et al., 2009). Moreover, because our analyses included tests of significance of within-person indirect effects, and the distribution of indirect effects is skewed in most cases, we used the Bayesian estimator with default (non-informative) priors and means for point estimates in our analyses.

## Results

### Preliminary analyses

Means, standard deviations, Cronbach's alphas, day-level variance, and zero-order correlations of study variables are shown in Table 1. As indicators of internal consistency, Multilevel Cronbach's alphas (Geldhof et al., 2014) were calculated (refer to columns 5 and 6 in Table 1). Internal consistency was relatively low, but still acceptable for sleep quality (between-person level α = 0.84; within-person level α = 0.71), and good to excellent for all other scales (between-person level α ≥ 0.95; within-person level α ≥ 0.80).

**Table 1:** Means, standard deviations, Cronbach's alphas, day-level variance, and zero-order correlations of study variables.

	***M^a^***	***SD^a^***	***SD^b^***	**α^c^**	**α^d^**	**1-ICC^e^**	**1**	**2**	**3**	**4**	**5**	**6**	**7**	**8**	**9**
1 Sleep quality	4.10	0.50	0.62	0.84	0.71	60%	–	**0.12**	0.06	0.04	0.05	0.09	0.01	−0.01	−0.07
2 Occupational self-efficacy	4.01	0.61	0.46	0.99	0.87	37%	**0.29**	–	0.00	0.06	−0.03	0.07	−0.04	−**0.11**	0.00
3 Time pressure	2.39	0.81	0.94	0.95	0.90	57%	−0.08	−0.08	–	0.06	0.03	**0.16**	**0.16**	0.06	−0.05
4 Problem solving	2.75	0.89	0.85	0.95	0.80	48%	0.15	**0.24**	**0.28**	–	0.09	**0.37**	0.05	−**0.14**	−**0.21**
5 Planning and decision-making	3.56	0.86	0.95	0.99	0.92	55%	−0.01	**0.54**	−0.04	**0.25**	–	**0.18**	−0.05	.03	0.05
6 Challenge appraisal	2.66	0.77	0.82	0.96	0.86	53%	**0.27**	**0.33**	**0.27**	**0.45**	**0.42**	–	−0.09	−**0.17**	−**0.18**
7 Hindrance appraisal	1.72	0.61	0.66	0.96	0.84	54%	−**0.27**	−**0.59**	0.18	−0.02	−**0.37**	−0.15	–	**0.18**	0.06
8 Self-regulation effort	2.15	0.67	0.79	0.96	0.83	58%	0.04	−**0.48**	**0.37**	0.05	−**0.25**	−0.09	**0.31**	–	**0.40**
9 Workplace procrastination	1.68	0.61	0.57	0.97	0.87	47%	0.04	−**0.45**	**0.24**	−0.04	−0.13	−0.08	**0.30**	**0.68**	–

*Correlations below the diagonal are correlations at the between-person level (110 individuals). Correlations above the diagonal are correlations at the within-person level (762 day-level datasets). Numbers in bold indicate p < 0.05 for between- and within-person correlations*.

a *Means and standard deviations at the between-person level*.

b *Standard deviations at the within-person level*.

c *Multilevel Cronbach's alphas at the between-person level*.

d *Multilevel Cronbach's alphas at the within-person level*.

e *1-ICC, Percentage of variance at the within-person level; ICC, variance at the between-person level/(variance at the within-person level + variance at the between-person level)*.

Before testing our hypotheses, we also examined the degree of within-person and between-person variation in our data. There was substantial within-person variation, ranging between 37% (for occupational self-efficacy) and 60% (for sleep quality), calling for a multilevel approach to data analysis (refer to column 7 in Table 1).

### Hypotheses testing

We tested both our hypotheses simultaneously in a single Bayesian MSEM, controlling for sleep quality and occupational self-efficacy. Specifically, challenge appraisal and hindrance appraisal were regressed on both control variables as well as all three work characteristics; self-regulation effort was regressed on both control variables, the three work characteristics, and the two cognitive appraisals; and workplace procrastination was regressed on both control variables, the three work characteristics, the two cognitive appraisals, and self-regulation effort. We allowed for correlations among control variables and work characteristics as well as between challenge appraisal and hindrance appraisal. The model specification was the same for both levels of analysis. With a posterior predictive *p* value of 0.460 being close to the ideal value of 0.500 (Muthén and Asparouhov, 2012, p. 315), the model showed an excellent model fit.

On the within-person level results from the Bayesian MSEM (shown in Table 2) revealed consistent positive (i.e., favorable) relationships of all three work characteristics with challenge appraisal. Although time pressure shared a positive (i.e., adverse) relationship with hindrance appraisal at the within-person level, neither problem solving nor planning and decision-making were related to hindrance appraisal within persons. Both cognitive appraisals predicted self-regulation effort within persons: Challenge appraisal reduced self-regulation effort (i.e., a favorable effect), whereas hindrance appraisal increased self-regulation effort (i.e., an adverse effect). As expected, self-regulation effort shared a positive (i.e., adverse) relationship with workplace procrastination. Results also revealed that problem-solving also had a direct negative (i.e., favorable) effect on workplace procrastination. Overall, the model explained significant but rather small portions of variance in both cognitive appraisals, self-regulation effort, and workplace procrastination on the within-person level (see Table 2). This might be because measurement errors usually affect the lower level of analysis in multilevel models.

**Table 2:** Results from Bayesian MSEM analysis.

	**Challenge appraisal**			**Hindrance appraisal**			**Self-regulation effort**			**Workplace procrastination**		
		**Bayesian 95% CI**			**Bayesian 95% CI**			**Bayesian 95% CI**			**Bayesian 95% CI**	
	**Estimate**	**LL**	**UL**	**Estimate**	**LL**	**UL**	**Estimate**	**LL**	**UL**	**Estimate**	**LL**	**UL**
Between-person level (*R*^2^)	**0.426**	0.237	0.603	**0.420**	0.232	0.606	**0.454**	0.254	0.642	**0.541**	0.369	0.695
Intercept	−1.277	−2.915	0.346	**4.381**	3.026	5.756	2.228	−0.069	4.504	0.449	−1.455	2.280
Sleep quality	**0.377**	0.025	0.728	−0.164	−0.456	0.115	0.354	−0.011	0.716	0.157	−0.140	0.462
Occupational self-efficacy	0.027	−0.324	0.377	−**0.514**	−0.807	−0.222	−**0.617**	−0.986	−0.253	−0.253	−0.585	0.082
Time pressure	**0.228**	0.013	0.448	0.060	−0.122	0.246	**0.314**	0.107	0.538	0.034	−0.156	0.224
Problem solving	**0.211**	0.005	0.413	0.089	−0.078	0.252	0.060	−0.132	0.244	−0.048	−0.203	0.109
Planning and decision-making	**0.325**	0.075	0.583	−0.091	−0.300	0.114	0.106	−0.156	0.381	0.141	−0.072	0.358
Challenge appraisal							−0.141	−0.318	0.340	−0.024	−0.204	0.328
Hindrance appraisal							0.016	−0.401	0.114	0.062	−0.233	0.180
Self-regulation effort										**0.513**	0.274	0.750
Residual variance	**0.393**	0.258	0.559	**0.258**	0.159	0.386	**0.293**	0.183	0.440	**0.205**	0.138	0.290
Within-person level (*R*^2^)	**0.188**	0.126	0.252	**0.042**	0.014	0.081	**0.096**	0.049	0.152	**0.214**	0.154	0.280
Sleep quality	0.078	−0.041	0.196	0.002	−0.104	0.106	0.019	−0.100	0.140	−0.057	−0.138	0.025
Occupational self-efficacy	0.067	−0.088	0.222	−0.058	−0.194	0.079	−0.149	−0.308	0.014	0.082	−0.028	0.192
Time pressure	**0.111**	0.038	0.184	**0.109**	0.044	0.173	0.045	−0.037	0.126	−0.031	−0.087	0.025
Problem solving	**0.333**	0.252	0.413	0.037	−0.032	0.110	−0.096	−0.193	0.002	−**0.086**	−0.155	−0.019
Planning and decision-making	**0.121**	0.048	0.194	−0.044	−0.107	0.020	0.054	−0.029	0.134	0.043	−0.013	0.099
Challenge appraisal							−**0.125**	0.083	0.313	−0.051	−0.074	0.085
Hindrance appraisal							**0.200**	−0.231	−0.018	0.005	−0.121	0.021
Self-regulation effort										**0.272**	0.212	0.332
Residual variance	**0.564**	0.493	0.641	**0.433**	0.379	0.492	**0.584**	0.514	0.661	**0.267**	0.235	0.302

*Table shows unstandardized estimates; CI, credibility interval, LL, lower limit, UL, upper limit; Numbers in bold indicate that the estimate is significant at α = 0.05 level based on Bayesian 95% CI*.

On the between-person level results from the Bayesian MSEM (shown in Table 2) again revealed consistent positive (i.e., favorable) relationships of all three work characteristics with challenge appraisal. Additionally, sleep quality also shared a positive (i.e., favorable) relationship with challenge appraisal on the between-person level. There were no relationships between any of the three work characteristics with hindrance appraisal on the between-person level. However, results indicated a negative (i.e., favorable) relationship between occupational self-efficacy and hindrance appraisal between persons. Neither challenge appraisal nor hindrance appraisal predicted self-regulation effort at the between-person level. However, occupational self-efficacy shared a negative (i.e., favorable) relationship with self-regulation effort, whereas time pressure shared a positive (i.e., adverse) relationship with self-regulation effort between persons. Finally, self-regulation effort shared a positive (i.e., adverse) relationship with workplace procrastination on the between-person level. Overall, the model explained significant and comparably large portions of variance in both cognitive appraisals, self-regulation effort, and workplace procrastination on the between-person level (see Table 2).

Hypotheses 1a–c predicted negative (i.e., favorable) within-person serial indirect effects of work characteristics on workplace procrastination via increased challenge appraisal and consequently decreased self-regulation effort. In line with Hypotheses 1a–c, results revealed negative (i.e., favorable) within-person serial indirect effects of all three work characteristics on workplace procrastination via challenge appraisal and self-regulation effort (refer to Table 3). Thus, Hypotheses 1a–c were supported.

**Table 3:** Within-person serial indirect effects from Bayesian MSEM with credibility intervals.

		**Bayesian 95% CI**	
	**Estimate**	**LL**	**UL**
**Serial indirect effects via challenge appraisal (CA) and self-regulation effort (SRE)**		
Time pressure → CA → SRE → workplace procrastination	−**0.004**	−0.009	−0.000
Problem solving → CA → SRE → workplace procrastination	−**0.011**	−0.023	−0.002
Planning and decision-making → CA → SRE → workplace procrastination	−**0.004**	−0.009	−0.000
**Serial indirect effects via hindrance appraisal (HA) and self-regulation effort (SRE)**		
Time pressure → HA → SRE → workplace procrastination	**0.006**	0.002	0.012
Problem solving → HA → SRE → workplace procrastination	0.002	−0.002	0.007
Planning and decision-making → HA → SRE → workplace procrastination	−0.002	−0.007	0.001

*Table shows unstandardized within-person estimates; CI, credibility interval; LL, lower limit; UL, upper limit. Numbers in bold indicate that the estimate is significant at α = 0.05 level based on Bayesian 95% CI*.

Hypotheses 2a–c predicted positive (i.e., adverse) within-person serial indirect effects of work characteristics on workplace procrastination via increased hindrance appraisal and consequently increased self-regulation effort. In line with Hypothesis 2a, results revealed a positive (i.e., adverse) within-person serial indirect effect of time pressure on workplace procrastination via hindrance appraisal and self-regulation effort (refer to Table 3). However, contrary to Hypotheses 2b and 2c, the respective within-person serial indirect effects were not significant (refer to Table 3). Thus, although Hypothesis 2a was supported, Hypotheses 2b and 2c had to be rejected.

### Additional analysis

Given that occupational self-efficacy showed rather high correlations with some of the other variables in the model, we decided to also run an additional analysis without control variables to check whether the results of our analysis are stable. The model specification was identical to the one used in the main analysis apart from removing sleep quality and occupational self-efficacy from the model on both levels of analysis. The posterior predictive *p* value of 0.455 was again close to the ideal value of 0.500 (Muthén and Asparouhov, 2012, p. 315). Thus, the model also showed an excellent model fit. The results of these Bayesian MSEM can be found in Supplementary Materials.

The results from this additional analysis are largely comparable with the main analysis performed for hypotheses testing. On the within-person level, the pattern of significance of results in the additional analysis was identical to the main analysis with one exception: The effect of problem solving on self-regulation effort fell short of significance in our main analysis but was significant in the additional analysis (compare Supplementary Table 1). On the between-person level, the pattern of significance of results in the additional analysis was again identical to the main analysis with three exceptions: Time pressure was no longer significantly related to challenge appraisal, planning and decision-making became a predictor of hindrance appraisal, and the intercept of self-regulation effort became significant in the additional analysis (compare Supplementary Table 1).

In sum, most differences in the statistical significance of individual paths and intercepts were on the between-person level that was of less interest in our study. On the within-person level there was only a minor change and, more importantly, all serial indirect effects that were previously significant stayed significant and all serial indirect effects that were previously not significant remained to be not significant (compare Supplementary Table 2). Thus, although the statistical significance of some individual paths and intercepts changed between the main analysis and the additional analysis, the conclusions we draw for our hypotheses do not change when removing the control variables from the analysis.

## Discussion

Our diary study showed that work characteristics are linked to workplace procrastination via within-person processes of cognitive appraisal and self-regulation. Of the three work characteristics investigated, only time pressure was linked to both challenge and hindrance appraisal on the within-person level. Problem solving as well as planning and decision-making triggered only challenge appraisal but not hindrance appraisal. However, we found the expected negative (i.e., favorable) serial indirect effects for all three work characteristics on workplace procrastination via challenge appraisal and self-regulation effort. Further, the results also revealed a positive (i.e., adverse) serial indirect effect of time pressure on workplace procrastination via hindrance appraisal and self-regulation effort for time pressure. This results show that self-regulation effort to overcome inner resistances increases not only when employees perceive their work situation as more hindering on a specific workday, but also when they feel less challenged.

Our findings also showed that problem solving and planning and decision-making were only positively related with challenge appraisal (and shared no relationship with hindrance appraisal). Previous research rather consistently reported simultaneous positive relationships of challenge stressors with both, challenge appraisal and hindrance appraisal (e.g., Searle and Auton, 2015). This finding could suggest that not all challenge stressors necessarily also trigger hindrance appraisals and that it might make sense to further differentiate challenge stressors into those that trigger both, challenge appraisal and hindrance appraisal, and thus could be labeled ambivalent/mixed challenge stressors (e.g., time pressure), and those that trigger only challenge appraisal and thus could be labeled consistent/pure challenge stressors (e.g., problem solving, planning and decision making).

Our results also show that challenge and hindrance appraisal affected self-regulation effort only on the within-person level. On the between-person level self-regulation effort was strongly related with occupational self-efficacy in a favorable way. Still, on both levels, self-regulation effort was strongly related to workplace procrastination. This indicates that cognitive appraisals of the work situation play a relevant role for situation-specific self-regulation effort and daily workplace procrastination. In contrast, on the between-person level, occupational self-efficacy played an important role for persistent self-regulation effort and chronic workplace procrastination. This illustrates that, depending on the level of analysis and the perspective one takes, different variables seem to play a role in the emergence of workplace procrastination (compare Klingsieck, 2013).

In our study, sleep quality did not affect workplace procrastination, neither on the within- nor on the between-person level. We controlled for sleep quality as other studies on workplace procrastination (Kühnel et al., 2016, 2018) indicated that sleep is necessary to restore energy and willpower and thus plays a role in the emergence of workplace procrastination. An explanation for not finding effects of sleep quality on workplace procrastination could be that we used a different measure for sleep quality. In contrast to previous studies that used only a single item to measure sleep quality, we decided to adapt four items from the Insomnia Severity Index (Bastien et al., 2001). It may be that the beneficial effects of a good night's sleep are better measured with the single item used in previous research. An alternative explanation could be that Kühnel et al. (2016, 2018) have also shown that the effects of poor sleep quality on workplace procrastination increase with the circadian misalignment of sleep-wake preferences and work times. Thus, if the circadian misalignment in our sample was small, this could also explain why we did not find any effects of sleep quality on workplace procrastination.

Overall, our study shows that by taking a situational perspective on procrastination scholars may gain additional insights on the within-person processes that explain the emergence of procrastination. In line with the results of a limited number of previous diary studies, our study demonstrated that procrastination has meaningful fluctuations on the day level that can be explained by situational factors. Coming from a work and organizational psychology background, we focused on work characteristics as situational factors that potentially trigger both favorable and unfavorable cognitive appraisals of the work situation. To our knowledge, our study was also the first study to show that cognitive appraisals have an effect on employees' self-regulation effort and that higher levels of self-regulation effort translate themselves into higher levels of workplace procrastination. We think that it might be interesting to check whether analogous within-person processes also explain the emergence of procrastination in other settings.

### Strengths, limitations, and avenues for future research

A major strength of our study is that its diary study design allowed us to investigate within-person processes that link work characteristics to workplace procrastination in daily working life. Hence, our study is among the first studies investigating situational factors as antecedents of procrastination in work settings. By asking participants to fill out diary entries at three measurement occasions per workday, we were also able to measure several variables at separate points in time and thus reduce concerns about potential common-method bias (Podsakoff et al., 2003).

Moreover, we conducted additional analysis to test whether and how the inclusion of sleep quality and occupational self-efficacy as control variables affected our results. We included these control variables because we expected them to have an impact on within-person processes that explain intra-individual fluctuations in workplace procrastination across workdays. Although, our results suggest that sleep quality and occupational self-efficacy do not have a relevant impact on within-person processes that link work characteristics to workplace procrastination, they also showed that our control variables were able to explain between-person differences in workplace procrastination. Most importantly, the additional analysis revealed that the conclusions we draw from our analysis regarding our hypotheses are the same whether we include sleep quality and occupational self-efficacy in our models or not.

A limitation of our study is that—despite the three measurement occasions—not all measures could be separated in time. We decided to measure control variables separately from work characteristics and cognitive appraisals, which were again measured separately from of self-regulation effort and workplace procrastination. However, this means that we are not able to causally interpret the relationships between variables measured at the same point in time (i.e., work characteristics and cognitive appraisals respectively self-regulation effort and workplace procrastination). Future research might want to increase the number of measurements per day even further and implement within-day cross-lagged designs to allow stronger inferences about causality. However, it should be kept in mind that increasing the number of measurement occasions per workday might negatively impact on participants willingness to participate and their compliance in a diary study.

Another limitation of our diary study is that we were only able to gather self-report data from our participants. It would be desirable for future research to also obtain ratings on job stressors and/or performance from supervisors or colleagues. It should be noted, however, that asking supervisors and/or colleagues to submit such ratings over a period of multiple workdays also comes along with problems that might result in an increase in missing values. It might happen that participants do not have any contact with their supervisor or a specific colleague on a given workday and thus the supervisor or colleague would not be able to submit valid ratings. Moreover, the supervisor or colleague might forget to submit their ratings or even decide to drop out of the study. As a consequence diary studies in organizational research usually do not obtain ratings from supervisors or colleagues (for examples see Binnewies et al., 2009; Parke et al., 2018).

It should also be noted that we decided to measure workplace procrastination with a scale from Tuckman (1991) that had previously been adapted to measure workplace procrastination in diary studies (Kühnel et al., 2016, 2018). Although this measure is not typically used in research on procrastination outside of the work context (where procrastination is also oftentimes investigated from a differential psychology perspective rather than a situational perspective), we think that our measure of workplace procrastination is well-suited for our study design. However, future research might also want to adapt the recently developed workplace procrastination scale by Metin et al. (2016) or other well-validated procrastination scales (cf. Svartdal and Steel, 2017) for the use in diary studies on workplace procrastination.

Future research could also aim to better integrate the situational and differential psychology perspectives on procrastination. It could be interesting to investigate how between-person differences might impact on the within-person processes that lead to workplace procrastination. For example, future research might want to analyze whether the processes differ between individuals depending on their stress mindset (Crum et al., 2013; Casper et al., 2017). It seems reasonable to assume that individuals with a stress-is-enhancing mindset might appraise time pressure more as a challenge than as a hindrance and thus they might require less self-regulation effort when working under time pressure and consequently procrastinate less than individuals with a stress-is-debilitating mindset.

It might also be interesting to dig deeper into the within-person processes that link work characteristics to workplace procrastination on a daily level. Future research could measure affect and affect regulation alongside cognitive appraisals and self-regulation effort. This might enable researchers to control for affect and affect regulation in their analyses or even investigate whether or not the results would be comparable to those obtained in the present study.

Finally, future research on workplace procrastination might also want to devote itself to further integrate the literature on workplace procrastination with other streams of work and organizational psychology literature, like the literature on withdrawal behavior (cf. van Eerde, 2016), task completion (e.g., Claessens et al., 2010), and online media use at work (e.g., Syrek et al., in press). We have the impression that although procrastination is not quite often investigated in work settings, there are many related topics in work and organizational psychology that might benefit from a better inclusion of procrastination research and vice versa.

## Conclusion

Our study shows that it is important to investigate procrastination also in non-academic contexts as well as from a situational perspective, because not all findings might be transferable from the academic context to other contexts respectively from the person level to the day level. Because workplace procrastination generates enormous costs for both, individuals and organizations, we encourage other researchers to help further advance scholarly knowledge by investigating workplace procrastination in their future studies.

## Author contributions

All authors contributed to the conception and design of the study. OW and KH organized the collection and preparation of data. RP performed the statistical analyses, wrote the first draft of the manuscript, and also made changes to the manuscript during the interactive review stage. All authors have read and edited the manuscript and suggested improvements at several stages during the preparation and revision of the manuscript.

### Conflict of interest statement

The authors declare that the research was conducted in the absence of any commercial or financial relationships that could be construed as a potential conflict of interest.
