# Supplementary material for: Procrastination in Daily Working Life: A Diary Study on Within-Person Processes That Link Work Characteristics to Workplace Procrastination
Source: Front Psychol. 2018 Jul 5;9:1087. doi: 10.3389/fpsyg.2018.01087 (PMC6042014; doi:10.3389/fpsyg.2018.01087)
Supplement: Supplementary file 1 [file Table_1.docx]

Supplementary Material

Procrastination in Daily Working Life:
A Diary Study on Within-Person Processes that Link
Work Characteristics to Workplace Procrastination

Roman Prem*, Tabea E. Scheel, Oliver Weigelt, Katja Hoffmann, Christian Korunka

*** Correspondence:** Dr. Roman Prem: roman.prem@univie.ac.at

# Supplementary Table

The tables on the following page show results from Bayesian MSEM without controlling for sleep quality and occupational self-efficacy.

Table 1. Results from alternative Bayesian MSEM analysis

|  | Challenge appraisal | | |  | Hindrance appraisal | | |  | Self-regulation effort | | |  | Workplace procrastination | | |
| --- | --- | --- | --- | --- | --- | --- | --- | --- | --- | --- | --- | --- | --- | --- | --- |
|  |  | Bayesian 95% CI | |  |  | Bayesian 95% CI | |  |  | Bayesian 95% CI | |  |  | Bayesian 95% CI | |
|  | Estimate | LL | UL |  | Estimate | LL | UL |  | Estimate | LL | UL |  | Estimate | LL | UL |
| Between level (*R*^2^) | **.340** | .164 | .525 |  | **.182** | .040 | .365 |  | **.271** | .105 | .459 |  | **.502** | .321 | .665 |
| Intercept | 0.328 | -0.609 | 1.237 |  | **2.287** | 1.480 | 3.107 |  | **1.675** | 0.561 | 2.775 |  | 0.096 | -0.814 | 1.001 |
| Time pressure | 0.194 | -0.025 | 0.413 |  | 0.125 | -0.071 | 0.324 |  | **0.288** | 0.072 | 0.508 |  | -0.004 | -0.186 | 0.170 |
| Problem solving | **0.261** | 0.062 | 0.461 |  | 0.013 | -0.165 | 0.193 |  | 0.036 | -0.168 | 0.239 |  | -0.069 | -0.219 | 0.082 |
| Planning and decision-making | **0.322** | 0.110 | 0.536 |  | **-0.256** | -0.441 | -0.068 |  | -0.104 | -0.339 | 0.132 |  | 0.073 | -0.106 | 0.251 |
| Challenge appraisal |  |  |  |  |  |  |  |  | 0.175 | -0.107 | 0.458 |  | 0.115 | -0.102 | 0.328 |
| Hindrance appraisal |  |  |  |  |  |  |  |  | -0.092 | -0.349 | 0.161 |  | 0.005 | -0.186 | 0.197 |
| Self-regulation effort |  |  |  |  |  |  |  |  |  |  |  |  | **0.612** | 0.412 | 0.814 |
| Residual variance | **0.440** | 0.302 | 0.605 |  | **0.354** | 0.234 | 0.492 |  | **0.372** | 0.249 | 0.537 |  | **0.214** | 0.144 | 0.303 |
| Within level (*R*^2^) | **.181** | .119 | .248 |  | **.035** | .009 | .072 |  | **.088** | .043 | .143 |  | **.205** | .143 | .271 |
| Time pressure | **0.114** | 0.039 | 0.186 |  | **0.106** | 0.041 | 0.172 |  | 0.050 | -0.033 | 0.135 |  | -0.031 | -0.088 | 0.024 |
| Problem solving | **0.338** | 0.257 | 0.422 |  | 0.037 | -0.035 | 0.108 |  | **-0.103** | -0.202 | -0.005 |  | **-0.082** | -0.150 | -0.015 |
| Planning and decision-making | **0.123** | 0.049 | 0.195 |  | -0.043 | -0.108 | 0.020 |  | 0.062 | -0.020 | 0.142 |  | 0.043 | -0.013 | 0.098 |
| Challenge appraisal |  |  |  |  |  |  |  |  | **0.204** | 0.087 | 0.319 |  | 0.000 | -0.079 | 0.077 |
| Hindrance appraisal |  |  |  |  |  |  |  |  | **-0.131** | -0.234 | -0.025 |  | -0.055 | -0.129 | 0.018 |
| Self-regulation effort |  |  |  |  |  |  |  |  |  |  |  |  | **0.268** | 0.209 | 0.328 |
| Residual variance | **0.562** | 0.488 | 0.641 |  | **0.434** | 0.382 | 0.492 |  | **0.586** | 0.518 | 0.662 |  | **0.268** | 0.237 | 0.302 |

*Note*. Table shows unstandardized estimates;
CI = credibility interval, LL = lower limit, UL = upper limit;
Numbers in bold indicate that the estimate is significant at α *=* .05 level based on Bayesian 95% CI.
Numbers that are underlined indicate that significance at α *=* .05 level based on Bayesian 95% CI differs from Table 2 (in the main article).
