# Supplementary material for: Procrastination in Daily Working Life: A Diary Study on Within-Person Processes That Link Work Characteristics to Workplace Procrastination
Source: Front Psychol. 2018 Jul 5;9:1087. doi: 10.3389/fpsyg.2018.01087 (PMC6042014; doi:10.3389/fpsyg.2018.01087)
Supplement: Supplementary file 2 [file Table_2.docx]

Supplementary Material

Procrastination in Daily Working Life:
A Diary Study on Within-Person Processes that Link
Work Characteristics to Workplace Procrastination

Roman Prem*, Tabea E. Scheel, Oliver Weigelt, Katja Hoffmann, Christian Korunka

*** Correspondence:** Dr. Roman Prem: roman.prem@univie.ac.at

# Supplementary Table

The tables on the following page show results from Bayesian MSEM without controlling for sleep quality and occupational self-efficacy.

Table 2. Within-person serial indirect effects from alternative Bayesian MSEM with credibility intervals

|  |  |  | Bayesian 95% CI | |  |
| --- | --- | --- | --- | --- | --- |
|  | Estimate |  | LL | UL |  |
| Serial indirect effects via challenge appraisal (CA) and self-regulation effort (SRE) |  |  |  |  |  |
| Time pressure → CA → SRE → workplace procrastination | **-0.004** |  | -0.009 | -0.000 |  |
| Problem solving → CA → SRE → workplace procrastination | **-0.012** |  | -0.023 | -0.002 |  |
| Planning and decision-making → CA → SRE → workplace procrastination | **-0.004** |  | -0.010 | -0.001 |  |
| Serial indirect effects via hindrance appraisal (HA) and self-regulation effort (SRE) |  |  |  |  |  |
| Time pressure → HA → SRE → workplace procrastination | **0.006** |  | 0.002 | 0.012 |  |
| Problem solving → HA → SRE → workplace procrastination | 0.002 |  | -0.002 | 0.007 |  |
| Planning and decision-making → HA → SRE → workplace procrastination | -0.002 |  | -0.007 | 0.001 |  |

*Note*. Table shows unstandardized within-person estimates;
CI = credibility interval, LL = lower limit, UL = upper limit;
Numbers in bold indicate that the estimate is significant at α *=* .05 level based on Bayesian 95% CI.
Significance at α *=* .05 level based on Bayesian 95% CI does not differ from Table 3 (in the main article) for any of the estimates.
